# POPs Contamination of Aquatic Sediments of the Cerna River Watershed, Romania—Prevalence, Pollution Sources, and Ecological Risk Assessment

**DOI:** 10.3390/toxics14100908

**Published:** 2026-10-14

**Authors:** Irina Monica Simó, Vlad Alexandru Pănescu, Mihaela Cătălina Popovici, Ye Yang, Jérome Vial, Maria Concetta Bruzzoniti, Mihail Simion Beldean-Galea

**Affiliations:** 1 Faculty of Environmental Science and Engineering, Babeș-Bolyai University, 30 Fântânele street, 400294 Cluj-Napoca, Romania; irina.simo@ubbcluj.ro (I.M.S.); ye.yang@ubbcluj.ro (Y.Y.); 2 Raluca Ripan Institute for Research in Chemistry, Babeș-Bolyai University, 30 Fântânele street, 400294 Cluj-Napoca, Romania; mihaela.herghelegiu@ubbcluj.ro; 3 Key Laboratory of Recycling and Eco-Treatment of Waste Biomass of Zhejiang Province, School of Environmental and Natural Resources, Zhejiang University of Science and Technology, Hangzhou 310023, China; 4 École Supérieure de Physique et de Chimie Industrielle de la Ville de Paris, Paris Sciences et Lettres University, 10 Rue Vauquelin, 75231 Paris, France; jerome.vial@espci.psl.eu; 5 Department of Chemistry, University of Turin, via P. Giuria 5, 10125 Turin, Italy; mariaconcetta.bruzzoniti@unito.it

**Keywords:** persistent organic pollutants (POPs), aquatic sediments, ultrasound-assisted extraction (USAE), gas chromatography (GC), biological risk, Cerna River basin

## Abstract

This study aims to evaluate the prevalence and pollution sources of polycyclic aromatic hydrocarbons (PAHs), polychlorinated biphenyls (PCBs), and organochlorine pesticides (OCPs) in sediments of the Cerna River and its main tributaries, and to assess the associated ecological risk on aquatic ecosystem. The compounds were extracted from the dried sediments by ultrasound-assisted extraction and analyzed by gas chromatography coupled with mass spectrometry for PAHs, and with electron capture detector for PCBs and OCPs. Six isomeric and molecular ratios were used to identify the sources of pollution, three for PAHs, and three for OCPs. Ecological risk was assessed using the effects-range low (ERL) and effects-range median (ERM) methods, and health risk using toxic equivalent factors (TEFs). Twenty sediment samples from ten sampling points in two campaigns (October 2023 and August 2024) were collected. Results revealed high concentrations of ƩPAHs, with concentrations between 3.86 and 4450.75 ng/g dw, with values of the isomeric ratios indicating pyrogenic origins. OCPs concentrations ranged between 4.25 and 129.56 ng/g dw. PAHs presented a low biological risk, having concentrations below ERL. Low biological risk was also observed for ƩPCBs, ƩDDTs with values below ERL, and some between ERL and ERM, indicating occasional possible adverse effects.

## 1. Introduction

Persistent organic pollutants (POPs) are a group of chemical compounds that are characterized by high toxicity, resistance to degradation, bioaccumulation, and long-range transport capacity [1]. These compounds can persist in the environment for long periods of time and tend to accumulate in sediments, soil, and aquatic or terrestrial organisms, therefore having negative effects on human health and ecosystems [2]. The most studied classes of POPs are polycyclic aromatic hydrocarbons (PAHs), organochlorine pesticides (OCPs), and polychlorinated biphenyls (PCBs).

Polycyclic aromatic hydrocarbons (PAHs) are mainly generated by the incomplete combustion of fossil fuels, biomass, and waste, and due to their high chemical stability, widespread distribution in the environment [3], and their demonstrated carcinogenic, mutagenic, and teratogenic potential [4,5,6]. These compounds are included on the list of priority contaminants of the United States Environmental Protection Agency (EPA) [7,8,9]. PAHs have been detected in multiple environmental compartments, including air [10], sediment [11], dust [12], water [13], and soil [14]. PAHs have a strong affinity for solid particles in the environment, favoring accumulation in soil and sediment.

Organochlorine pesticides (OCPs) are a class of POPs consisting of various groups of compounds, and are very resistant to biological and chemical degradation, an aspect which made them to intensively used for decades in agriculture as insecticides, bactericides, fungicides, herbicides, and even rodenticides [15]. The most commonly used compounds include HCHs (hexa-chloro-cyclohexanes), DDTs (dichlorodiphenyl trichloroethane), endosulfan, chlordane, and chlorinated cyclodienes (aldrin, dieldrin, endrin).

HCHs are solid and synthetic compounds with four common isomers, namely *α*, *β*, *γ*, and *δ*-HCH. Of these, γ-HCH (also known as Lindane) has been the most widely used insecticide in crop protection. Although its use has been banned for decades, it is still used in some countries to treat scabies and lice [16]. *γ*-HCH isomer is considered the most toxic of the class, therefore many studies have focused on it, neglecting the other isomers, which is suboptimal, since all isomers can be found in the environment [17]. From an acute exposure perspective, γ-HCH is the most toxic to mammals, followed by *α*-, *δ*-, and *β*- HCH. In the case of chronic exposure, the *β*- isomer presents the highest level of toxicity, followed by *α*-, *γ*-, and *δ*-HCH [17].

DDT (dichlorodiphenyl trichloroethane) was developed in the late 1930s and is one of the best known modern synthetic insecticides. Due to its effectiveness, it was widely used in agriculture, forestry, and malaria control in the 20th century [18]. Like other persistent organic pollutants, DDT has a high bioaccumulation capacity, it can be transported over long distances and is of major concern for human and ecological health. Its common metabolites include *4,4′*-DDE, *4,4′*-DDD, and methoxychlor. Of these, *4,4′*-DDE and *4,4′*-DDD share similar characteristics but are considered less toxic than the parent compound [19].

Endosulfan is an organochlorine insecticide, composed of *α*- and *β*- isomers, frequently produced as a mixture (30% to 70% ratios) and has been widely used for the control of biting or stinging pests (caterpillars, aphids, thrips) [20].

Chlordane and its derivative compounds have been used extensively in the past for the control of termites and various soil pests, being classified as persistent OCPs and endocrine disruptors [21].

Aldrin, dieldrin, and endrin are also part of the same class of highly toxic and persistent organochlorine insecticides, used predominantly between the 1950s and 1970s [22].

Heptachlor is a chlorinated cyclodiene used for soil pest control, which can be oxidized to heptachlor epoxide, and is considered more toxic and persistent than its parent compound [23].

Polychlorinated biphenyls (PCBs), were previously used in electrical equipment, capacitors, and transformers, are extremely resistant to degradation, tend to bioaccumulate, and can cause neurotoxic effects and endocrine disruption [24].

Due to these hazardous characteristics, POPs are regulated by the Stockholm Convention (2001), which initially banned 12 substances, expanding the list to 28 compounds by 2022 [25]. The transport of POPs occurs through air, water, and soil, allowing contamination of remote regions to reach others non-polluted, and with their chemical persistence, makes them difficult to eliminate from the environment.

At the European-Union level, the legislative framework that establishes water policy is [26], which states that a good ecological status of a water body is achieved if both the chemical and biological status are good. If we consider that the benthos lives in the upper layers of sediment, the degree of contamination of the sediments will directly influence the biological status of the water body [27]. For this reason, in order to have a good ecological status, it is necessary to also have good sediment quality, which is why special attention has been paid to sediment quality in recent times. This has required the development of different sediment quality standards [28,29,30] aimed at assessing the ecotoxicological risk of sediments on biota.

For the assessment of the ecotoxicological potential of sediment contamination, the sediment quality guidelines system proposed by [31] can be used, which proposes for the mixture of PAH, OCPs, and PCBs relative to dry sediment with 10% TOC (total organic carbon) two limit thresholds, namely TEC (threshold effect concentration)—no adverse effects, and PEC (probable effect concentration)—probable adverse toxic effects. The most common method for assessing the biological risk is the one proposed by [32], which uses the effects-range approach (ERL/ERM), having two threshold levels, namely effects-range low (ERL), and effects-range median (ERM), used to determine the impact of individual compounds from sediments and the adverse biological effects it has on microorganisms.

These biological risk assessment methods are frequently accompanied by other studies used to identify pollution sources [33,34,35,36,37,38], aiming not only to pinpoint potential sources of pollution but also to enable the implementation of measures to reduce pollution and improve the quality of water bodies. The assessment of the distribution and biological risk involved of these pollutants are determined by the analysis of sediments, water, or other living organisms, using methods such as ultrasound-assisted extraction and gas chromatography coupled with mass spectrometry (GC–MS) or electron capture detector (GC-ECD) [14,35,36,37,38,39,40].

Across this study, the contamination with POPs of the Cerna River basin was investigated, the main objectives being the following: (1) determination of the concentration level of PAHs, OCPs, and PCBs in sediments; (2) to determine the distribution of these compounds along the Cerna River and its tributaries; and (3) to assess the temporal variations of contamination between the two sampling campaigns. Therefore, twenty sediment samples were collected from ten sampling points located along the Cerna River and its tributaries, within the framework of two monitoring campaigns. All samples have been analyzed for POPs using GC–MS and GC-ECD, with the obtained results being compared between the sampling points and between the two investigated periods in order to highlight the distribution of the contaminants and the changes in contamination levels over time and in different areas of the Cerna River basin. The obtained data was used to estimate the ecological risk associated with these compounds in the studied area, as a necessity imposed for alignment with the “One Health” concept.

## 2. Materials and Methods

### 2.1. Description of the Study Area, Sampling Points, and Sediment Sampling Procedure

**Figure 1:**
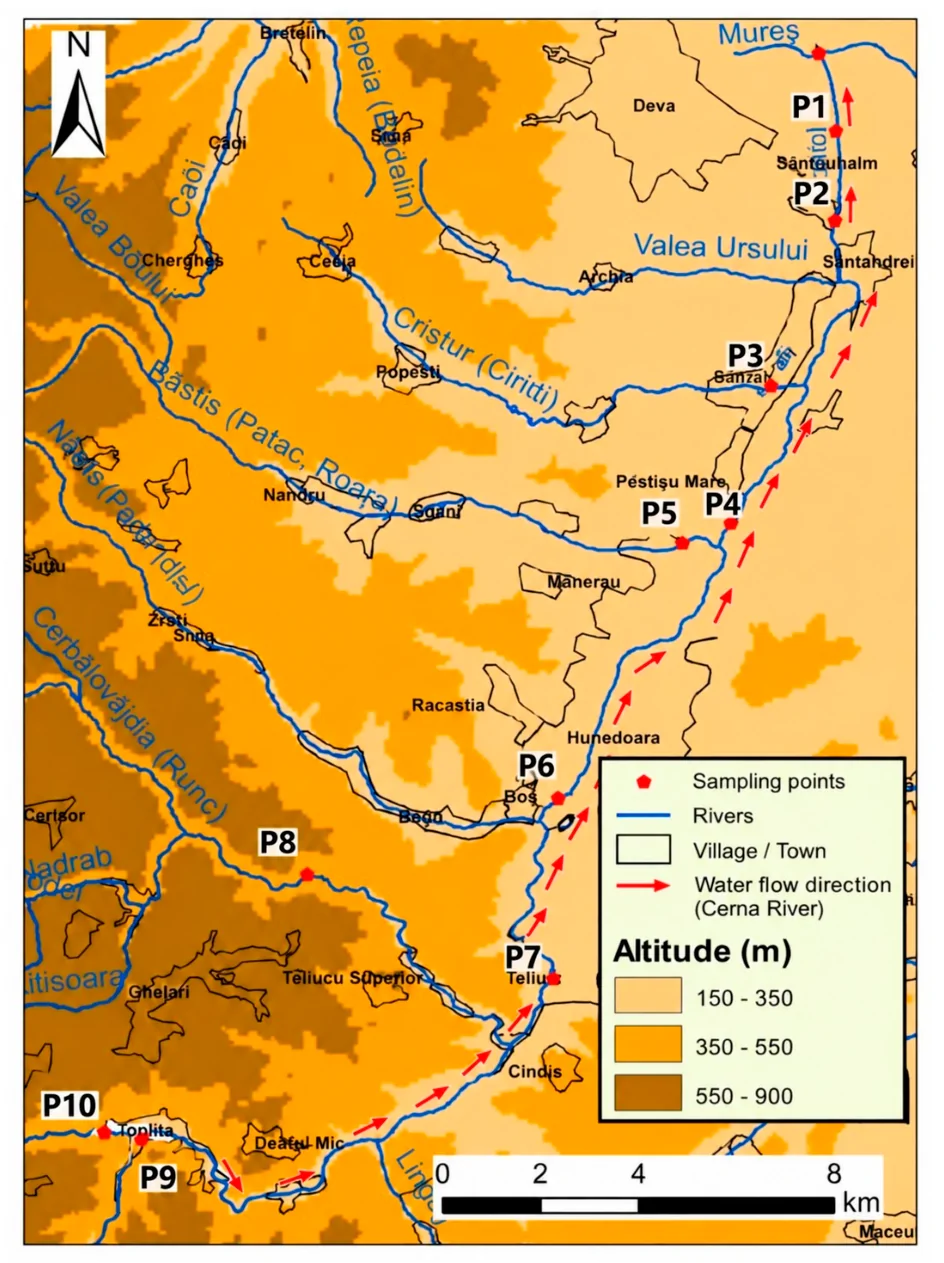
Map of the studied watershed and location of the sampling points.

The research focused on the Cerna River basin, the main hydrographic basin of Hunedoara, an area with intense mining and metallurgical activity. Cerna River has a length of 73 km from its source to its confluence with Mureş River, a catchment area of 727 km^2^ and 11 registered tributaries (courses with a length of at least 5 km, and a catchment area of at least 10 km^2^): Cernuşoriţa, Ireanc, Negoi, Preajba, Bunila (Bilea), and Vălăriţa—upstream of the Cinciş dam, Govăjdia (Runc), Zlaşti, Peştiş (Petac, Roata), Cristur (Cîrjiţi), Valea Ursului—downstream of the Cinciş dam. To study the impact of anthropogenic activities on the sediment quality in the Cerna River basin, 10 sampling points located at the confluence of the main tributaries with the Cerna River were selected (Figure 1). The name, geographical positioning and GIS coordinates of the sampling points are given in the Supplementary Materials Table S1.

A total of twenty sediment samples were collected in the two campaigns, the first campaign taking place in early October (2023) and the second campaign in late August (2024) from selected sampling points, to study the evolution of sediment quality over time.

The two sampling periods were chosen taking into account the hydrological regime in the autumn season which correspond to the relatively constant period of the hydrological regime in the study area.

Sediment sampling was carried out at each of the ten selected monitoring points by collecting three distinct samples in the watercourse section (edges and middle), which were subsequently unified into a single sample. A telescopic sampling instrument equipped with a plastic sampling cup was used for sediment collection, allowing sampling without excessive disturbance of the substrate near the sampling point. At each monitoring point, the sampling device was carefully lowered into the water column and positioned at the level of the sediment layer, followed by its introduction into the sediment and the collection of representative samples from a depth ranging from 5 to 12 cm. This depth range was selected to target sediments considered relevant for the assessment of contaminant accumulation in the aquatic environment. Sampling was carried out carefully to minimize sediment resuspension and loss of fine fraction during the process. After sampling, the sediment collected in the cup was carefully transferred to a polypropylene (PP) container intended for sediment sample storage. The containers were sealed immediately after sample collection and individually labeled with the necessary identification information, namely the sampling point code, sampling time and sampling campaign details, to ensure traceability during subsequent preparation and analysis steps. After collection, the sediment samples were transported to the laboratory in a refrigerated bag, where they were subsequently subjected to preparation steps for further analysis.

### 2.2. Chemicals and Reagents

The PAHs standard solution was purchased from Restek (Restek Corporation, Bellefonte, PA, USA) and contains 16 priority congeners such as naphthalene (NAP), acenaphthylene (ACY), acenaphthene (ACE), fluorene (FL), phenanthrene (PHE), anthracene (ANT), fluoranthene (FLT), pyrene (PYR), benz[*a*]anthracene (BaA), chrysene (CRY), benzo[*b*]fluoranthene (BbF), benzo[*k*]fluoranthene (BkF), benzo[a]pyrene (BaP), dibenz[*a,h*]anthracene (DBahA), benzo[*g,h,i*]perylene (BghiP), and indeno[*1,2,3-cd*]pyrene (IPYR) (CRM EPA Method 8310 PAH Mixture, Supelco, Merck Romania SRL, Bucharest, Romania) in concentration of 500 μg mL^−1^ of each compound in acetonitrile: toluene (92:8 *v*/*v*).

OCPs standard solution was purchased from Supelco (Merck Romania SRL, Bucharest, Romania) in a concentration of 2000 μg mL^−1^ in hexane: toluene (1:1 *v*/*v*) (EPA CLP Organochlorine Pesticide Mix, Supelco, Merck Romania SRL, Bucharest, Romania) having the following 22 compounds as components: tecnazene, *α*-HCH, hexachlorobenzene, *β*-HCH, γ-HCH, quintozene, heptachlor, aldrin, heptachlor epoxide, heptachlor endo-epoxide, trans-chlordane, *4,4’*-DDT, *2,4’*-DDT, *4,4’*-DDE, *2,4’*-DDE, *4,4’*-DDD, *2,4’*-DDD, *α*-endosulfan, *β*-endosulfan, dieldrin, endrin, methoxychlor.

Regarding PCBs, a mixture containing 12 PCBs was used, which contained PCB *-18*, *-28*, *-31*, *-52*, *-44*, *-101*, *-114*, *-149*, *-153*, *-138*, *-180*, *-194*, in a concentration of 10 μg/mL in heptane, purchased from Supelco (Merck Romania SRL, Bucharest, Romania).

The extract purification was performed using silica, alumina, and granular anhydrous sodium sulfate (200–300 mesh; Merck, Darmstadt, Germany), which were all activated for 6 h at 450 °C in a furnace.

Desulphurization of the extract was performed using copper of <425 μm size and 99.5% purity (Sigma-Aldrich, Merck, Germany), activated using hydrochloric acid (1 N).

Acetone, *n*-hexane, and dichloromethane were all in HPLC grade purity, being acquired from Merck (Darmstadt, Germany), while the milli-Q water which was used was prepared using a Milli-Q-Plus ultra-pure water system from Millipore (Milford, MA, USA).

### 2.3. Instrumental Analysis of POPs

The analysis of persistent organic pollutants was performed in two steps. By gas chromatography coupled with mass spectrometry for the analysis of PAHs, and gas chromatography coupled with an electron capture detector for the analysis of OCPs and PCBs according to [40].

Thus, for the analysis of PAHs, a Focus gas chromatograph (Thermo Electron Corporation) equipped with a DSQII mass spectrometer and a TriPlus autosampler was used. The separation of the compounds was performed using a DB-5 MS capillary column (25 m × 0.25 mm i.d., 0.25 µm film thickness, Thermo), with a temperature gradient starting at 60 °C for 1 min, followed by an increase from 60 to 130 °C at 10 °C/min. Subsequently, the temperature was raised from 130 to 300 °C at 3 °C/min and an isothermal hold of 5 min at 300 °C.

The carrier gas was helium (99.999%), with a flow rate of 1.2 mL/min, the inlet temperature was set at 310 °C, transfer line at 300 °C, and the ion source temperature at 200 °C. The ionization voltage was −70 eV, and data acquisition was done in SIM (Selected Ion Monitoring) mode using Xcalibur software (v3.0.63, Thermo Electron Corp.).

Regarding OCPs and PCBs, the analysis was performed via GC-ECD using a Trace GC equipped with a ^63^Ni electron capture detector, and a TriPlus Autosampler (Thermo Electron Corporation, Waltham, MA, USA). Separation of the two classes was done using a capillary column model HP-5MS (30 m × 0.25 mm i.d., 1.0 μm film thickness, Agilent, Santa Clara, CA, USA). The temperature gradient program started from 70 °C to 180 °C at a rate of 25 °C/min, from 180 to 200 °C at 1 °C/min, from 200 to 260 °C at a rate of 2 °C/min, and from 260 to 300 °C at 5 °C/min with a holding time of 5 min at 300 °C. The temperatures of the inlet and detector were set at 270 °C and 300 °C, respectively.

The carrier gas used for this method was nitrogen of 99.999% purity, having a constant flow rate of 2 mL/min. The make-up gas was nitrogen, having a flow rate of 40 mL/min.

The identification of OCPs and PCBs was based on the retention times of the standard mixtures, using the selected molecular ions specific to each compound using Chrom-Card software (2.14, Thermo Fisher Scientific), while quantification was done based on internal standards in accordance with our previous publication (Barrhoumi et al., 2019, [36]).

### 2.4. Sediment Sample Processing, Extraction, and Purification of POPs

The sediment samples were dried at room temperature to remove moisture, then crushed and homogenized using a mortar and pestle to obtain a homogeneous sample. Subsequently, the resulting sample was sieved through a 63 µm sieve. The sieved material was collected and transferred to polyethylene bags, appropriately labeled, and stored at 4 °C until further processing.

The extraction and purification procedure for the POPs was realized in accordance with the method developed by Barrhoumi et al., 2019 [35], but with minor changes. Approximately eight grams of dried and homogenized sediments were placed in a 50 mL centrifuge tube. A total of 20 mL of acetone: *n*-hexane (1:1, *v*/*v*) was added, followed by a 20-min ultrasonation. The resulted mixture was centrifuged at 4400 rpm for five minutes, then transferred to a glass vial. This procedure was repeated two more times with fresh solvent; the three obtained extracts being combined to form a final extract. To ensure that there were no traces of sulfur in the sample, one gram of activated copper was added to the extract, being left overnight for desulfurization. The following day, 10 mL of n-hexane was added, and the copper was removed by decantation, the final extract being concentrated to approximately two milliliters.

The purification step was performed using a glass chromatography column with an inner diameter of 15 mm, which was successively filled with three grams of activated silica, three grams of activated alumina, and one gram of dehydrated Na_2_SO_4_, in this order. The column was previously conditioned with 20 mL of *n*-hexane, followed by loading the concentrated extract onto the column. The target compounds were eluted first with 20 mL of *n*-hexane, followed by 40 mL of *n*-hexane: dichloromethane (80:20, *v*/*v*). All portions from the elution were combined and concentrated to approximately 2 mL, then evaporated completely under a stream of nitrogen and re-dissolved in a final volume of 0.2 mL in the same mixture of acetone: hexane (1:1, *v*/*v*).

This method guarantees efficient extraction and rigorous purification of persistent organic pollutants, desulfurization with active copper, and the use of a silica-alumina-Na_2_SO_4_ column to eliminate interferences, thus preparing samples for precise analysis. The extraction and analyses procedure were validated in a previous study [36] using materials of certified reference, such as EC-3 (PAHs, PCBs, and OCPs in marine sediments from Niagara River in Lake Ontario), with the obtained results being in accordance with the necessary requirements for this type of analyses.

### 2.5. Risk Assessment

#### 2.5.1. Ecological and Biological Risk Assessment Interpretation

Ecological and biological risk assessment is a valid method of estimating the toxic effect of several types of pollutants present in lake sediments which can represent a risk on lake organisms. The determination of ecological risk regarding sediment contamination is performed using procedures such as sediment quality guidelines (SQGs), a method which has been used for several years in order to identify potential ecological and toxicological risks on microorganisms [41]. This approach was chosen as it is accessible to all environmental agencies, as contamination is based on certain concentrations of pollutants that fall within parameters of maximum allowed limits [42].

The method which is most often used is the effects-range approach (ERL/ERM), developed in order to determine the impact of individual chemicals from sediments and the adverse biological effects that it has on microorganisms [32].

The effects were determined using percentile and each chemical and chemical group has two final indices. The first one is the lower 10th percentile data in which effects for each chemical are signaled, referred to as effects-range low (ERL), and the second indices are the 50th percentile of observed effects, named effects-range median (ERM). These two values are used to estimate the concentrations of certain pollutants in which effects were observed and are divided into three levels [32]:

− values below ERL results have a low probability of ecological risk, with effects rarely occurring (less than 10%);
− values between ERL and ERM result in effects occasionally occurring;
− values greater than ERM result in effects frequently occurring (more than 50%), having negative impact.

In addition to the effects-range approach, the mean effect-range median quotient (m-ERM-Q) was used, being another approach in assessing pollution and possible biological effects. The base of this second approach is the assumption that all the contaminants have already been tested with the effects-range approach and have the same toxicity and act as acute and chronic toxins. This method is newer than the effects-range approach (ERL/ERM) and it has certain limitations, but it is yet another very used guideline for sediment toxicity, and it has a series of advantages by comparing it to the previous used method as it makes interpretations of complex mixtures of pollutants rather than individual contaminants offering numerical index based on the degree of contamination of sediment samples (Table 1) [43].

#### 2.5.2. Health Risk Assessment Interpretation

For the PAHs health risk assessment, the Toxic Equivalency Factor approach (TEF) was used. The principle of this approach is to separate the studied hydrocarbons into carcinogenic and noncarcinogenic groups and to offer them a carcinogenic factor using BaP as an indicator of toxicity and as a surrogate compound for all the carcinogenic PAHs [45].

Benzo[*a*]pyrene equivalent concentrations (BaP_Eq_) and Benzo[*a*]pyrene toxic equivalency factors (TEQ) are used for assessing the potential biological effects of PAHs on water microorganisms.

The TEQs have been calculated using the following equations:

BaP*_Eq_* = TEF*_i_* × C*_i_*, (1)

(2) $$\mathrm{TEQ}=\sum_{k=0}^{n}{\mathrm{BaP}}_{\mathrm{Eq}}$$

where [46]

- BaP*_Eq_* is the toxic equivalent concentration of an individual congener from the sample;
- TEF*_i_* is the BaP toxic equivalency factor for the compound *i*;
- C*_i_* is the measured concentration for compound *i*;
- TEQ is the toxic equivalent for a sample;
- *n* is the number of measured compounds representing the total TEQ.

Therefore, 16 PAHs are used, each of them being offered a toxic factor (TEF) to indicate the individual carcinogenic potency relative to BaP; thus, a TEF of 1.0 is applied to carcinogenic hydrocarbons and a TEF of 0 is applied to noncarcinogenic ones (Table S2) [45].

## 3. Results

### 3.1. Prevalence and Distribution of POPs in Sediments

A total of twenty sediment samples were collected in the two campaigns, the first campaign taking place in October (2023) and the second campaign in August (2024) from different locations in the Cerna River basin. The obtained results show that the concentration of POPs in sediments during the two campaigns have spatial and temporal variation in the 10 sampling points (Table 2).

The highest concentrations were recorded for PAHs with concentrations ranging from 3.86 and 14.65 ng/g dw in point P5 to 1139.23 and 4450.75 ng/g dw in point P9. High concentrations were also recorded in sampling points P3 (309.10 and 1145.24 ng/g dw) and P10 (129.99 and 514.36 ng/g dw). The high level of PAHs in sampling points P9 and P10 can be explained by the existence of a coal mining and charcoal production in the area, while the values in point P3 can be explained by the existence of an intense metallurgical activity. The temporal trend is one of decrease between the two sampling campaigns, the possible causes being related to weather conditions, a winter with the highest temperatures in the history of measurements carried out in Romania, which led to significantly lower combustion of fuels for heating homes, or the reduction of anthropogenic activities generating PAHs, such as the reduction of mining activities in the area and the closure of coal-fired power plants (Mintia coal-fired power plant), a process that began in 2022, respectively the closure of the metallurgical plant in Hunedoara (starting with 2021), measures taken by the Romanian Government as a necessity for alignment with the European Green Deal and for reducing the carbon footprint starting with 2022.

Regarding OCPs, the variations in concentrations are relatively small, with the exception of sampling point P3 which shows higher concentrations (72.82 ng/g dw in October 2023 and 129.56 ng/g dw in August 2024). The concentrations found are most likely the result of agricultural activities.

The lowest values were recorded for PCBs (below 41.01 ng/g dw), with higher values being recorded at points P3, P4, and P10, points located near industrial activities.

Total concentrations of POPs provide only partial information regarding their origin in environmental factors; therefore, a more detailed analysis of the results is necessary.

#### 3.1.1. PAH Results

**Figure 2:**
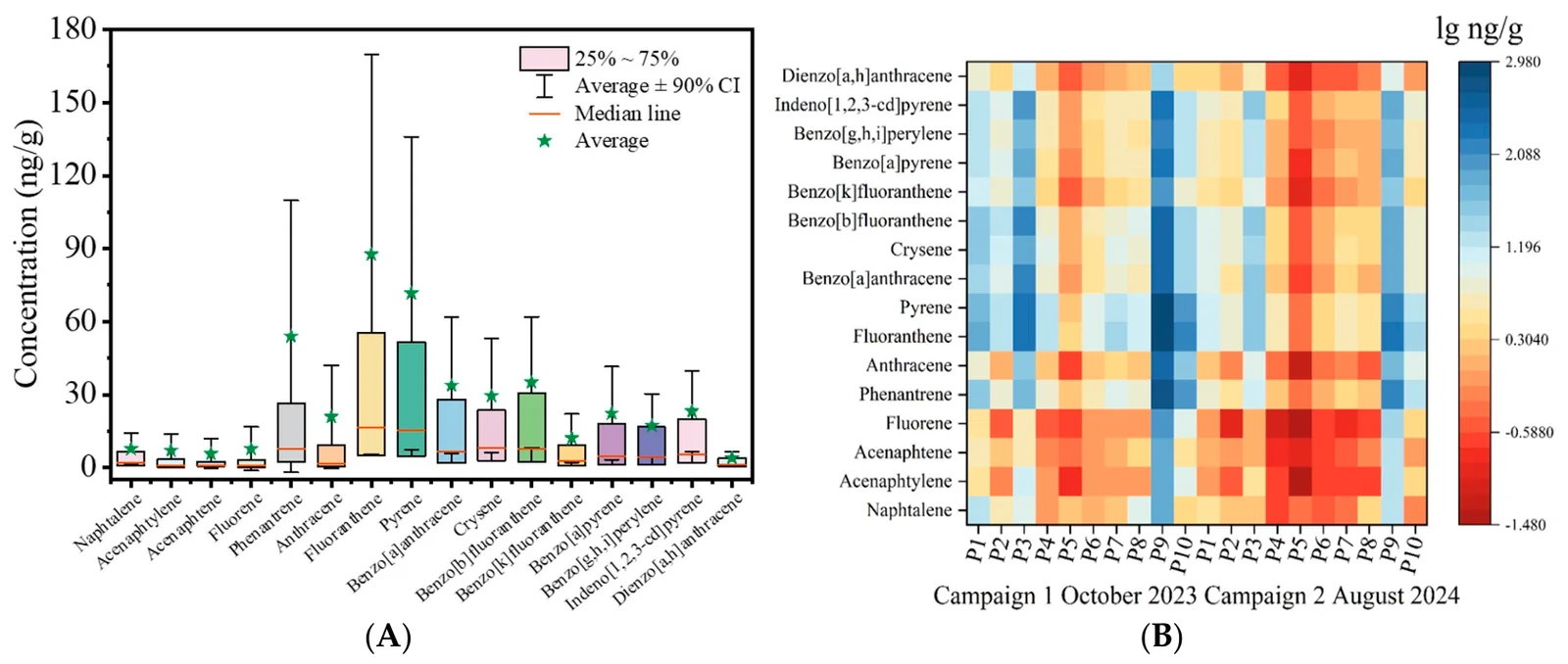
(**A**) Boxplots showing the concentration distributions of individual PAHs across the sampling sites. Boxes represent the interquartile ranges, orange lines indicate the medians, and green stars denote the mean concentrations; (**B**) heatmap illustrates the spatial and temporal variation in PAHs concentrations during Campaign 1 (October 2023) and Campaign 2 (August 2024). The data is presented as logarithmically transformed concentrations, lg[PAH]. Blue and red represent relatively high and low concentrations, respectively.

The concentration distributions differed substantially among the individual PAHs. The total PAHs concentrations range from 14.65 to 4450.75 ng/g dw in the October 2023 campaign (Table S3) with an average value of 696.3 ng/g and from 3.86 to 1139.23 ng/g dw in the August 2024 campaign (Table S4) with an average value of 182.34 ng/g dw. Most low-molecular-weight compounds exhibited relatively low concentrations and narrow interquartile ranges, indicating limited variation among sampling sites. In contrast, several higher-molecular-weight PAHs, particularly fluoranthene and pyrene, showed markedly higher concentrations and broader interquartile ranges. Their mean concentrations were considerably higher than their medians, suggesting right-skewed distributions and the presence of a few highly contaminated samples. Other compounds, including benzo[*a*]anthracene and chrysene, displayed intermediate concentration levels and variability. Overall, the boxplots indicate pronounced compound-specific differences in both PAH abundance and spatial heterogeneity (Figure 2A).

The heatmap revealed substantial temporal and compound-specific variability in PAH concentrations across the two sampling campaigns (Figure 2B). A pronounced high-concentration pattern was observed at Campaign 1, point P9, where several PAHs, particularly the high-molecular-weight compounds, exhibited dark-blue colors. This indicates a simultaneous increase in PAH concentrations at this sampling point. In contrast, relatively low concentrations were observed at Campaign 1, point P5, and several sampling points in Campaign 2, as reflected by the predominance of orange-to-red colors. The lower-molecular-weight PAHs, including naphthalene, acenaphthylene, acenaphthene, and fluorene, showed strong temporal fluctuations, with particularly low values during parts of Campaign 2. Higher-molecular-weight PAHs, such as benzo[*a*]pyrene, benzo[*b*]fluoranthene, benzo[*k*]fluoranthene, indeno [*1,2,3-cd*]pyrene, and dibenzo[*a,h*]anthracene, generally displayed more coherent variations across sampling points. Overall, the heterogeneous color patterns indicate that PAH concentrations were controlled by both compound-specific characteristics and time-dependent changes in local inputs, transport, and environmental conditions.

#### 3.1.2. OCPs Results

**Figure 3:**
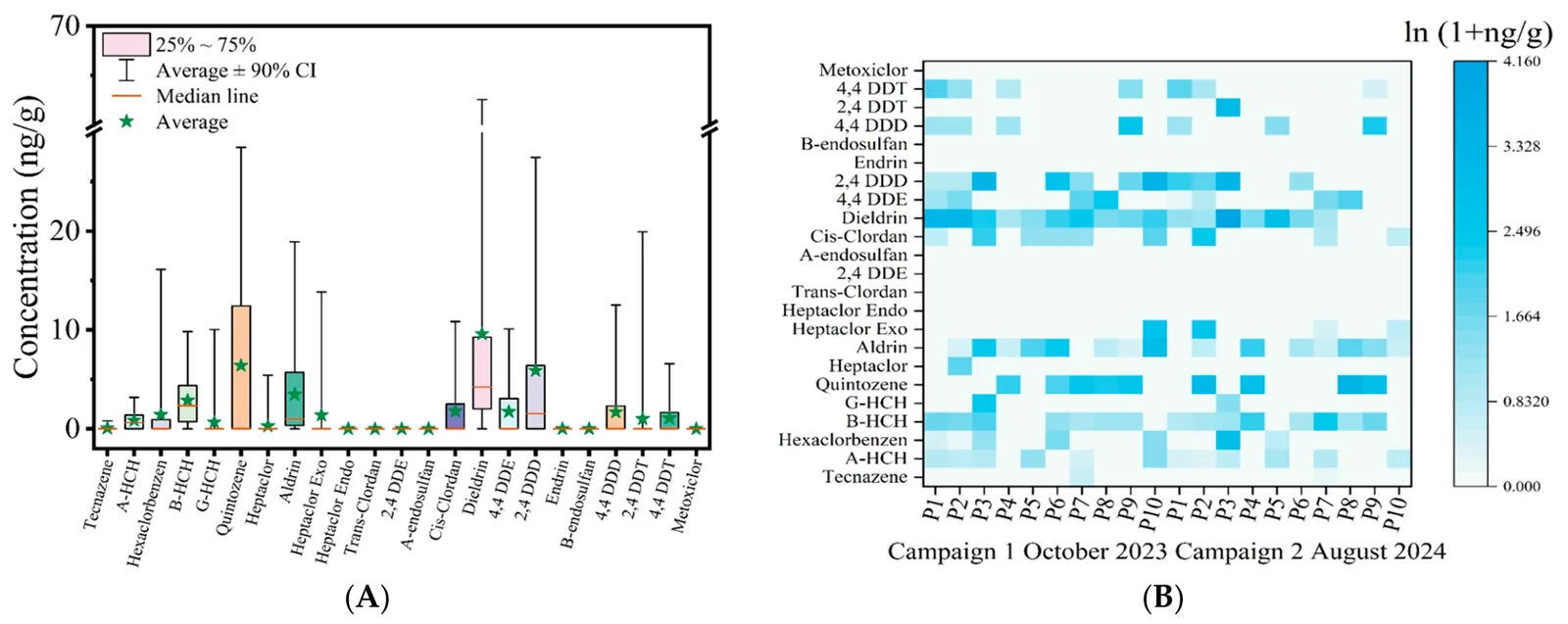
(**A**) Boxplots showing the concentrations of OCPs in samples collected. Boxes represent the interquartile ranges, orange lines indicate medians, and green stars denote means; (**B**) heatmap showing the spatial and temporal distribution of OCPs during Campaign 1 (October 2023) and Campaign 2 (August 2024). Color intensity represents the natural-log-transformed concentration, ln (1 + concentration).

OCPs concentrations range from not detected (nd) to 80.01 ng/g dw in the October 2023 campaign (Table S5) and from nd to 129.60 ng/g dw in the August 2024 campaign (Table S6). The concentration distributions of OCPs varied considerably among the analyzed compounds. Most OCPs showed low concentrations and relatively narrow interquartile ranges, indicating limited variability among sampling sites. In contrast, several compounds exhibited higher concentrations, broader boxes, and extended upper whiskers, suggesting greater spatial heterogeneity. For some OCPs, the mean concentrations were higher than the corresponding medians, indicating right-skewed distributions caused by a limited number of samples with elevated concentrations. Overall, the boxplots indicate compound-specific differences in the concentration levels and spatial variability of OCPs across the sampling sites (Figure 3A).

The heatmap revealed pronounced compound-specific and temporal variations in OCP concentrations between the two sampling campaigns (Figure 3B). Dieldrin showed the most widespread distribution and some of the highest concentrations, with elevated values detected across multiple sampling points. The DDT-related compounds, particularly *2,4’*-DDD and *4,4’*-DDT, also exhibited relatively high concentrations at several sampling points, although their distributions were spatially heterogeneous. Quintozene and β-HCH were detected at moderate-to-high levels across multiple sites, whereas several other compounds, including methoxychlor, endrin, trans-chlordane, and α-endosulfan, were generally absent or detected at very low concentrations. Hexachlorobenzene, heptachlor epoxide isomers, and *γ*-HCH showed localized increases at specific sampling sites. Overall, the heterogeneous color patterns indicate that OCP contamination was not uniformly distributed and may reflect differences in historical usage, persistence, transport, and site-specific environmental conditions.

#### 3.1.3. PCBs Results

**Figure 4:**
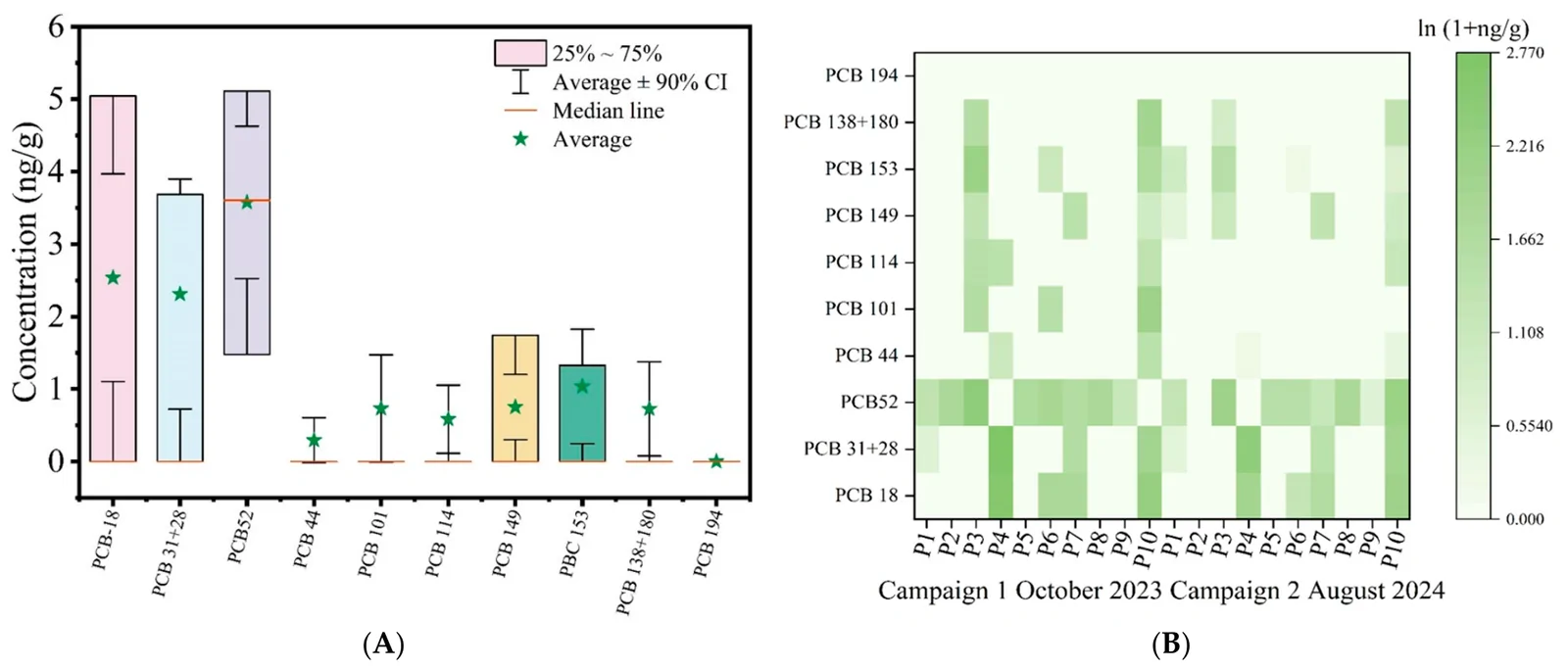
(**A**) Boxplots showing the concentration distributions of individual PCB congeners across the sampling sites. Boxes represent the interquartile ranges, orange lines indicate the medians, and green stars denote the mean concentrations; (**B**) heatmap showing the spatial and temporal distribution of selected PCB congeners during Campaign 1 (October 2023) and Campaign 2 (August 2024). Color intensity represents the logarithmically transformed concentration.

PCBs concentrations range from nd to 14.91 ng/g dw in the October 2023 campaign (Table S7) and from nd to 9.86 ng/g dw in the August 2024 campaign (Table S8). The PCB congeners displayed pronounced differences in both concentration levels and spatial variability. The first three congeners showed the highest concentrations and the widest interquartile ranges, indicating substantial heterogeneity among the sampling sites. Their mean values were clearly higher than the corresponding medians, suggesting strongly right-skewed distributions and the presence of samples with elevated PCB concentrations. The middle congeners generally occur at low concentrations, with medians close to the detection baseline. Moderate concentrations were observed for two additional congeners toward the middle-right of the plot, although their variability was lower than that of the dominant congeners. Overall, the results indicate that PCB contamination was concentrated on a limited number of congeners and sampling sites rather than being uniformly distributed across all samples (Figure 4A).

The heatmap revealed pronounced congener-specific and temporal variability in PCB concentrations across the two sampling campaigns (Figure 4B). PCB *-52* showed the most widespread occurrence, with relatively elevated levels detected across numerous sampling points in both campaigns, indicating its dominant contribution to the overall PCB profile. PCB *-31* + *28* and PCB *-18* also exhibited locally elevated concentrations, particularly at selected sampling points, but their distributions were less uniform. The higher-chlorinated congeners, including PCB *-138* + *180*, PCB *-153*, PCB *-149*, PCB *-114*, and PCB *-101*, were detected intermittently and generally at low-to-moderate levels. PCB 194 and PCB 44 were rarely detected and remained close to the lower end of the color scale. Several sampling points displayed simultaneous increases in multiple congeners, suggesting temporal changes in PCB inputs or environmental transport conditions. Overall, the heterogeneous distribution indicates that PCB contamination was dominated by a limited number of congeners, especially PCB *-52*, while the occurrence of the remaining congeners was more localized and variable.

### 3.2. Identification of Pollution Sources

In addition to determining the concentrations of POPs, identifying the sources of contamination is an essential aspect in assessing environmental quality, as it allows an integrated understanding of the origin, transport, and behavior of these compounds in aquatic and terrestrial ecosystems. Establishing the sources of contamination directly contributes to the foundation of effective pollution management strategies, facilitating the differentiation between natural and anthropogenic contributions, as well as the prioritization of emission control measures at local, regional and global levels. The presence of POPs in sediments and the water column is, in most cases, closely associated with anthropogenic activities, including industrial processes, fossil fuel combustion, the use of organochlorine pesticides in agriculture, as well as emissions from road traffic and intensive urbanization processes. Identifying the sources of contamination with POPs is an essential step in assessing environmental quality, which is why numerous analytical approaches have been developed to estimate the contribution of different sources.

#### 3.2.1. Source Identification of PAHs Using Diagnostic Ratios

Studies targeting the composition of polycyclic aromatic hydrocarbons demonstrate that there are variations in composition, aspect that depends greatly on their origin in the environment, which is often through combustion, a phenomenon used to establish their source and origin in the environment [33].

To better understand the use of these types of diagnostic ratios used in the determination of PAH sources, they have been classified into pyrogenic and petrogenic hydrocarbons, but they can also appear naturally in the environment [34].

These three types of sources represent the main categories of origin of PAHs in the environment, and diagnostic reports aim to establish the sources of emissions, pyrogenic PAHs being formed through pyrolysis processes, and when organic substances are exposed to high temperatures in conditions of limited or absent oxygen—usually between approximately 350 °C and over 1200 °C [47]. These processes include both anthropogenic activities such as the combustion of gasoline, diesel, or other types of oils, destructive distillation of coal, thermal cracking of petroleum residues, and unintentional processes such as incomplete combustion of fuels in engines, natural activities such as vegetation fires, incineration of wood and coal, and eruption of volcanoes [48,49]. Petrogenic PAHs on the other hand have their main sources attributed to spills of various oils, and several types of fossil fuel such as diesel, gasoline, and kerosene.

Three diagnostic ratios are used in this study, namely the ratio between light molecular weight PAHs and high molecular weight PAHs [∑LMW/∑HMW], the ratio between anthracene and the sum between anthracene and phenanthrene [ANT/(ANT + PHE)], and the ratio between fluoranthene and the sum between fluoranthene and pyrene [FLT/(FLT + PYR)].

- **∑LMW/∑HMW**

**Figure 5:**
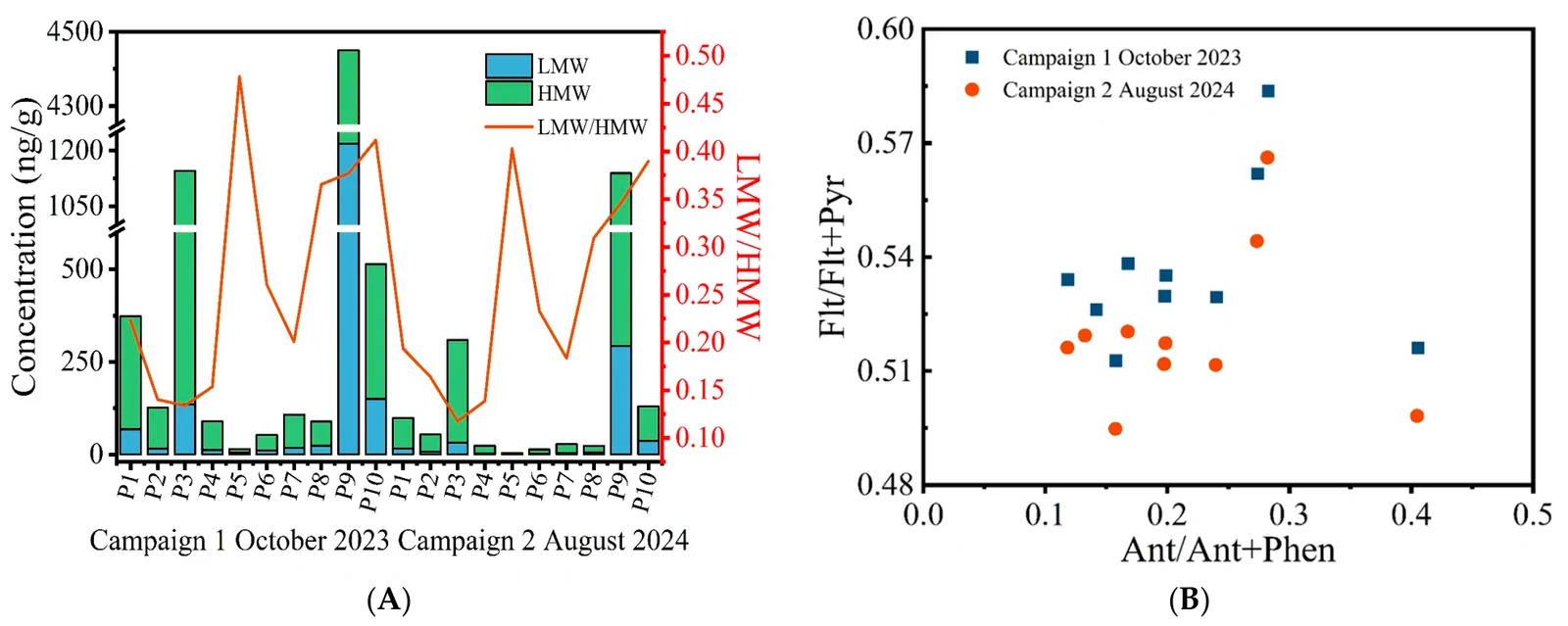
(**A**) Spatial variation in high and low molecular-weight PAHs and the **∑**LMW/**∑**HMW ratio across the sampling sites. Green and blue bars represent HMW-PAHs and LMW-PAHs, respectively, while the orange line indicates the **∑**LMW/**∑**HMW ratio; (**B**) PAH source diagnostic plot based on the molecular ratios Ant/(Ant + Phen) and Flt/(Flt + Pyr). Blue squares and orange circles represent the two sample groups, respectively.

One of the most common methods used for PAHs diagnostics consists in using of the 16 most widely spread PAHs, divided into two groups based on the number of aromatic rings they possess, such as low molecular weight (LMW) with two or three aromatic rings, most often of petrogenic origin, and high molecular weight (HMW) with four or more aromatic rings, often of pyrogenic origin [50,51]. A ratio lower or equal to <1 will indicate pyrogenic sources deriving from combustion processes and volcanoes, while a value higher than >1 will indicate petrogenic sources from fossil fuel, oil, and coal [35,52]. The results showed that PAHs in sediments have a pyrogenic source, as all the ratios are situated below one, with values ranging between 0.14 and 0.41 for the October 2023 (Table S3) campaign and between 0.12 and 0.40 for the August 2024 campaign (Table S4). The concentrations of HMW and LMW of PAHs varied considerably among the sampling sites. HMW-PAHs generally contributed more to the total PAH concentrations than LMW-PAHs, as indicated by the predominance of the green bars. The **∑**LMW/**∑**HMW ratio also showed substantial spatial variation, with relatively high values at sites sampling point P9 in October 2023 and low values at Sampling point P5 in August 2024. A high **∑**LMW/**∑**HMW ratio indicates a relatively greater contribution of LMW-PAHs, whereas a low ratio reflects the predominance of HMW-PAHs. The observed spatial heterogeneity suggests that the relative contributions of PAH sources varied among the sampling locations. Samples with relatively high **∑**LMW/**∑**HMW ratios may indicate stronger contributions from petroleum-related sources, whereas the predominance of HMW-PAHs and low **∑**LMW/**∑**HMW ratios are generally associated with combustion-related inputs (Figure 5A).

- **ANT/(ANT + PHE)**

The prevalence of phenanthrene over anthracene will often indicate that hydrocarbons will derive mainly from petrogenic activities, as phenanthrene is considered more thermodynamically stable than anthracene [34]. This ratio is often used to determine the dominance of combustion or petroleum sources, therefore, a value lower than <0.1 will indicate petrogenic source, and a value higher than >1 will indicate predominance of combustion source, mainly of pyrogenic origin [36].

The obtained results of the ANT/(ANT + PHEN) ratio range between 0.12 and 0.41 for the October 2023 campaign (Table S3), and between 0.12 and 0.40 for the August 2024 campaign, all of the samples having values above 0.1 (Table S4), suggesting that PAHs derive mainly from pyrogenic sources.

- **FLT/(FLT + PYR)**

Pyrene is considered to be a compound more stable than fluoranthene in terms of thermodynamics, so the presence of more pyrene will most likely indicate presence of petrogenic source, as it is found in raw crude oil, while fluoranthene is an indicator of pyrolysis processes [34].

The value this method uses is 0.5, as this is considered the sweet spot of the transition between petrogenic and combustion sources, and a ratio lower than <0.5 will indicate petrogenic origin, and a value higher than >0.5 will indicate pyrogenic origin [36].

Values of this ratio range between 0.51 and 0.58 for the October 2023 campaign (Table S3), and between 0.49 and 0.57 for the August 2024 campaign (Table S4). Thus, most of the samples indicate pyrogenic origin, all of the samples from 2023 indicate, while samples from 2024 campaign indicate pyrogenic origin for 8 out of 10 samples, as the remaining two indicate petrogenic origin.

For better evidence a cross-plot of Ant/(Ant + Phen) versus Flt/(Flt + Pyr) was used to diagnose the potential sources of PAHs (Figure 5B).

Most samples were distributed above the commonly used thresholds of 0.10 for Ant/(Ant + Phen) and 0.50 for Flt/(Flt + Pyr), indicating a predominantly pyrogenic contribution rather than a purely petrogenic source. Samples with Flt/(Flt + Pyr) values greater than 0.50 were consistent with contributions from the combustion of coal, wood, or other biomass, whereas samples falling within the range of 0.40–0.50 may reflect petroleum-combustion inputs. The relatively broad distribution of the samples suggests that PAHs may originate from mixed sources, with the relative contribution varying among sampling sites.

#### 3.2.2. Source Identification of OCPs Using Molecular Ratios

The DDT and HCH classes are the most widespread, given their heavy use in agriculture for decades. These classes have been used as technical mixtures with different percentages of the parent compound, and the use of molecular ratios between the parent compounds and their degradation products is useful to determine their source in the environment [15]. A lower level of the parent compound compared to its metabolites will indicate a sign of old use, while a higher level of the parent compound compared to its metabolites will indicate more recent input [53].

Three molecular ratios have been used, namely the ratio between the total amount of DDTs and HCHs (∑DDTs/∑HCHs), the ratio between the sum of the total amount of DDEs and DDDs and the total DDTs [(DDE + DDD)/∑DDTs], and the ratio between the -*α* and -*β* isomers (*α*-HCH/*β*-HCH) to determine the sources of OCPs (Tables S5 and S6). For the ratio **∑**DDTs/∑HCHs a higher percentage of DDT class will have a value above >1, while a value below <1 will indicate a higher presence of the HCH class [35]. The ratio (DDE + DDD)/∑DDTs is used to determine the time interval and usage of DDT, in order to determine if a certain matrix has undergone long term or recent usage of DDT [38]. DDT has usually been used in the form of technical DDTs, but it mostly comprises of a high percentage of *4,4’*- DDT. In the environment, a decrease in the DDT concentration corresponds to an increase in its metabolites of DDE and DDD [54]. The ratio uses 0.5 as the threshold value, with values higher than it suggesting historical input of DDT, while a value lower than 0.5 will indicate the presence of recent usage [37]. The ratio *α*-HCH/*β*-HCH is used to determine the origin of technical HCHs, using 11.8 as the threshold values. A value lower than 11.8 will determine the presence of technical HCH, indicating old use of HCHs, while a value higher than 11.8 will indicate a more recent input of HCHs [15,38].

For the ∑DDT/∑HCH ratio, the results of this study show a dominant presence of DDTs, as six samples from the October 2023 (Table S5) campaign have values greater than one, and only one shows the presence of HCHs, having values below one. The same situation was encountered for the August 2024 campaign (Table S6), where the presence of DDTs is dominant, and eight out of ten samples having values above one.

For the ratio (DDE + DDD)/∑DDTs, the results attest to a dominance of the historical DDT input, as most samples have values greater than 0.5 in eight samples from the October 2023 (Table S5) campaign and, respectively, nine samples from August 2024 (Table S6).

For the ratio *α*-HCH/*β*-HCH having scarce results from the two HCH isomers, only a few calculations could be performed, attesting old HCH input in both the campaigns, as all samples have ratios lower than the threshold value, with values between 0.29 and 0.35 for October 2023 (Table S5) and between 0.16 and 0.47 for the august 2024 campaign (Table S6).

### 3.3. Ecological and Biological Risk Assessment Results

Starting with the first campaign, both individual and total PAHs concentrations are lower than the recommended values of the effects-range low (ERL), having one exception for sample P9, corresponding to Toplița River, which has values higher than the ERL for eight compounds, as well for the total PAHs concentrations (Tables S3 and S4); therefore, from a PAHs-concentration perspective, these sediment samples do not represent an ecological risk [55].

Concentrations for OCPs and PCBs are scarce in comparison to PAHs, as the sediment quality guidelines (SQGs) only offer recommended values for *4,4*’-DDE, *4,4*’-DDD, *4,4*’-DDT, and ΣDDT [55]. All three of the DDT isomers have most of concentrations below the ERL, with a few samples having values higher than the ERL, while the concentration of the total DDTs and PCBs registered values above the ERL in nine out of ten samples (Table 3).

The second sampling campaign from August 2024 had even lower concentrations, with the majority of individual PAHs having concentrations lower than the ERL, and yet again sample P9 is the only sample with higher concentrations for acenaphthene and fluorene, which registers values above the ERL (Table 4).

PCBs are scarce, having the concentration below the ERL, while the DDT isomers register concentrations lower than the ERL in eight out of ten samples; the total DDTs concentration has values higher than the ERL in eight out of ten samples (Table 4).

The resulting concentrations for the three classes of POPs indicate a low ecological risk for the biota, with low possibility of adverse effects.

### 3.4. Health-Risk Assessment Results

The health-risk assessment was obtained using the toxicity of each individual PAHs for each sample, evaluated using the TEFs methods of benzo[*a*]pyrene equivalent concentrations (BaP_eq_) and benzo[*a*]pyrene toxic equivalency factors, often referred to as TEQs. The values of the previously mentioned methods for both sampling campaigns are provided in Table S9 for October 2023 campaign and Table S10 for August 2024 campaign.

The total concentrations of TEQ for the sum of PAHs (Σ_16_PAHs) ranges from 1.200 to 338.255 ng/g dw for October 2023, having a median of 12.13, and between 0.368 to 99.737 ng/g dw for August 2024, with a median of 4.33. The total TEQ concentrations of the seven carcinogenic PAHs (CRY, BbF, BkF, BaP, IPYR, DBahA, BghiP) [ΣCPAHs] range from 6.434 to 302.370 ng/g dw for October 2023, with a median of 11.25, and between 0.345 to 90.284 ng/g dw for August 2024, having a median of 4.04.

By making a comparison between the TEQ values of Σ_16_PAHs and ΣCPAHs, it resulted in a dominance of the PAHs of the carcinogenic aspect being the main contributors, representing 89.42% of the total TEQ for October 2023, and 90.58% for August 2024.

By comparing the TEQ of Σ_16_PAHs, ΣCPAHs, and the two most toxic PAHs, namely BaP DBahA, to the recommended values by the legislation (visible in Table 5), it can be confirmed that the concentrations detected in the two studies are well below normal thresholds, not representing a hazard for human health.

### 3.5. Comparison with Other Studies

In order to analyze the degree of contamination of the sediments in the study area, the results obtained were compared with other studies carried out worldwide. Thus, by analyzing the data presented in Table 6, it can be seen that they fall within the same concentration range for the sum of PAHs, OCPs, and PCBs both in other studies in Romania and studies in Italy, Turkey, and the USA. Higher values for PAHs, OCPs, and PCBs were recorded for the Fu River in China, these values being most likely related to the population density in the study area, which leads to higher values of the pollutants studied resulting from anthropogenic activities.

The values in the table show once again the importance of studying sediments and associated contaminants and represent a viable alternative in the absence of surface water monitoring data in order to establish water-source management programs, for better protection of aquatic ecosystems and human health.

## 4. Conclusions

To our knowledge, this is the first study that addresses the contamination of river sediments in Hunedoara County with these classes of compounds, introducing new aspects related to pollution sources and thus contributing to the international database on pollution with persistent organic pollutants.

According to the results obtained, the concentration of POPs in sediments during the two campaigns showed spatial and temporal variations in the 10 sampling points.

The highest values were recorded for PAHs, with concentrations ranging from a few ng/g to thousands of ng/g, the concentrations being influenced by the position of the sampling points and the anthropogenic activities. The isomeric ratios confirm a mining activity related to the production and exploitation of coal and the metallurgical industry carried out in the studied area. Thus, the values of the ∑LMW/∑HMW ratios indicate stronger contributions of combustion-related inputs compared to petroleum-related sources. The same conclusion emerges from the ANT/(ANT + PHE) and FLT/(FLT + PYR) ratios, which confirm a predominantly pyrogenic contribution rather than a purely petrogenic source.

OCP concentrations are relatively low, due to limited agricultural activity, with molecular ratios indicating predominantly the presence of DDT from historical uses.

PCB concentrations have values that are much lower than those of OCPs, with higher values of a few tens of ng/g being recorded in areas with metallurgical industry.

Regarding the ecological risk, the resulting concentrations for the three POP classes indicate a low ecological risk for biota, with a low possibility of adverse effects. However, if we refer to the concentrations detected in sediments in the two campaigns and compare them with the normal thresholds for soil provided for by Romanian legislation, they do not really represent a risk to human health. However, if we consider that 89.42% of the total TEQ for October 2023 and 90.58% for August 2024 is represented by PAH with carcinogenic potential, these values should constitute an alarm signal for public authorities responsible for surface-water management or environmental policy-making authorities in the formulation of environmental laws or regulations that introduce relevant standards for sediments that illustrate their toxicity for human health.

Finally, we can conclude that the present study fills the gap in data on sediment quality in the studied river basin, providing pertinent information on pollution sources and the risk associated with the studied POPs classes. The study confirms once again the usefulness of sediment analyses and the applicability of SQGs in ecological risk assessment.

## Figures and Tables

**Table 1:** Toxicity levels of m-ERM-Q values according to [44].

Range	Toxicity Level
m-ERM-Q ≤ 0.1	9% probability of toxicity
0.1 < m-ERM-Q ≤ 0.5	21% probability of toxicity
0.5 < m-ERM-Q ≤ 1.5	49% probability of toxicity
m-ERM-Q > 1.5	76% probability of toxicity

**Table 2:** Concentration of PAHs, OCPs, and PCBs in the two sampling campaigns.

Sampling Point	Total PAHs (ng/g)		Total OCPs (ng/g)		Total PCBs (ng/g)	
	October 2023	August 2024	October 2023	August 2024	October 2023	August 2024
P1	372.53	98.39	43.41	19.91	3.74	5.24
P2	126.57	53.95	47.90	58.69	5.23	0
P3	1145.24	309.10	72.82	129.56	31.47	14.31
P4	89.51	23.85	13.62	34.63	32.26	16.07
P5	14.65	3.86	14.90	23.15	4.71	3.68
P6	52.40	13.77	47.55	10.48	16.32	6.14
P7	107.82	27.92	37.32	20.37	16.48	12.40
P8	89.14	23.32	26.77	42.96	5.00	4.96
P9	4450.75	1139.23	40.83	32.97	2.06	0.89
P10	514.36	129.99	80.01	4.25	40.81	29.07

**Table 3:** Comparison of POPs concentration of October 2023 with the (ERL/ERM) guidelines and the (m-ERM-Q).

Compound	ERL ^a^	ERM ^a^	Concentration Ranges (ng/g dw)	Number of Samples			m-ERM-Q ^b^
				<ERL	ERL-ERM	>ERM	
**PAHs**							
Nap	*160*	*2100*	0.98–76.19	10	0	0	0.006
Acy	*44*	*640*	0.13–80.10	9	1	0	0.017
Ace	*16*	*500*	0.45–70.76	9	1	0	0.018
FL	*19*	*540*	0.20–103.24	9	1	0	0.023
Phe	*240*	*1500*	1.79–645.06	9	1	0	0.058
Ant	*85.3*	*1100*	0.24–243.46	9	1	0	0.031
Flu	*600*	*5100*	2.08–949.19	9	1	0	0.028
Pyr	*665*	*2600*	1.81–739.92	9	1	0	0.045
BaA	*261*	*1600*	0.76–307.86	9	1	0	0.033
Cry	*384*	*2800*	1.11–266.48	10	0	0	0.016
BaP	*430*	*1600*	0.56–215.95	10	0	0	0.022
DbahA	*63.4* ^c^	*260* ^c^	0.31–27.08	10	0	0	0.022
**ΣPAHs**	**4022**	**44,792**	**14.65–4450.75**	**9**	**1**	**0**	**0.016**
**PCBs**							
**ΣPCBs**	***22.7***	***180***	**2.06–40.81**	**9**	**1**	**0**	**0.052**
**OCPs**							
*4,4’*-DDE	*2.2*	*27*	<LOD–10.10	7	3	0	0.079
*4,4’*-DDD	*2.1*	*20*	<LOD–12.54	6	4	0	0.097
*4,4’*-DDT	*1.3*	*7.1*	<LOD–6.59	6	4	0	0.192
**ΣDDTs**	***1.6***	***46.1***	**<LOD–27.44**	**1**	**9**	**0**	**0.288**

^a^—values (ng/g dw) taken from [55]; ^b^—values (ng/g dw) taken from [44]; ^c^—estimated by [56]; ERL—effects-range low; ERM—effects-range median; m-ERM-Q—mean effect range median quotient; <LOD—lower than the detection limit.

**Table 4:** Comparison of POPs concentration of August 2024 with the (ERL/ERM) guidelines and the (m-ERM-Q).

Compound	ERL ^a^	ERM ^a^	Concentration Ranges (ng/g dw)	Number of Samples			m-ERM-Q ^b^
				<ERL	ERL-ERM	>ERM	
**PAHs**							
Nap	*160*	*2100*	0.21–16.48	10	0	0	0.001
Acy	*44*	*640*	0.03–20.00	10	0	0	0.004
Ace	*16*	*500*	0.12–19.22	9	1	0	0.005
FL	*19*	*540*	0.04–22.86	9	1	0	0.005
Phe	*240*	*1500*	0.43–155.90	10	0	0	0.014
Ant	*85.3*	*1100*	0.06–58.67	10	0	0	0.007
Flu	*600*	*5100*	0.45–205.29	10	0	0	0.006
Pyr	*665*	*2600*	0.42–171.94	10	0	0	0.010
BaA	*261*	*1600*	0.20–82.55	10	0	0	0.009
Cry	*384*	*2800*	0.35–82.56	10	0	0	0.005
BaP	*430*	*1600*	0.16–62.71	10	0	0	0.006
DbahA	*63.4* ^c^	*260* ^c^	0.11–9.29	10	0	0	0.008
**ΣPAHs**	**4022**	**44,792**	**3.86–1139.23**	10	0	0	0.004
**PCBs**							
**ΣPCBs**	***22.7***	***180***	**<LOD-29.07**	**10**	**0**	**0**	**0.052**
**OCPs**							
*4,4’*-DDE	*2.2*	*27*	<LOD–6.98	8	2	0	0.048
*4,4’*-DDD	*2.1*	*20*	<LOD–8.95	7	3	0	0.095
*4,4’*-DDT	*1.3*	*7.1*	<LOD–5.36	8	2	0	0.103
**ΣDDTs**	***1.6***	***46.1***	**<LOD–44.05**	**2**	**8**	**0**	**0.205**

^a^—values (ng/g dw) taken from [55]; ^b^—values (ng/g dw) taken from [44]; ^c^—estimated by [56]; ERL—effects-range low, ERM—effects-range median, m-ERM-Q—mean effect range median quotient; <LOD—lower than the detection limit.

**Table 5:** TEQ values comparison with legislative norms.

Compound	TEQ (ng/g dw)		Normal Value in Soils (ng/g dw) *	Threshold of Concern (ng/g dw) *
	October 2023	August 2024		
ΣPAHs	1.200–338.255	0.368–99.737	100	25,000
ΣCPAHs	1.114–302.370	0.345–90.284		
BaP	0.559–215.949	0.163–60.709	<20	5000

*—[57].

**Table 6:** The comparison of the obtained results with others obtained worldwide.

Country/River	∑PAHs * (ng/g dw)	∑OCPs * (ng/g dw)	∑PCBs * (ng/g dw)	References
Romania, Somes River	24.78–575.62	2.07–44.28	2.74–252.75	[35]
Romania, Olt River	21.62–601.02	12.59–93.28	9.98–279.42	[58]
Italy, Adriatic Sea	193–533	0.1–2.5	0.9–5.2	[59]
Turkey, Yeșilirmak River	53.33–594.49	4.25–27.75	0.70–1.15	[60]
China, Fu River	191.6–6360.5	5.4–707.6	4.2–197.8	[61]
USA, Colorado River	0.06–4.17	1.30–47.4	0.08–4.9	[62]
Romania, Cerna River	3.86–1139.23	4.25–129.56	3.74–40.81	This study

* ng/g dw.

## Data Availability

The original contributions presented in this study are included in the article/Supplementary Material. Further inquiries can be directed to the corresponding author.

## Supplementary Materials

The following supporting information can be downloaded at: https://www.mdpi.com/article/10.3390/toxics14100908/s1, Table S1: Geographical position of the sampling points; Table S2: TEFs for individual PAHs; Table S3: Total PAHs concentration (ng/g dw) and diagnostic ratios for the sources of PAHs for October 2023 campaign; Table S4: Total PAHs concentration (ng/g dw) and diagnostic ratios for the sources of PAHs for August 2024 campaign; Table S5: Total OCPs concentrations (ng/g dw) and molecular ratios for the sources of OCPs for October 2023 campaign; Table S6: Total OCPs concentrations (ng/g dw) and molecular ratios for the sources of OCPs for August 2024 campaign; Table S7: PCBs concentrations for October 2023 campaign; Table S8: PCBs concentrations for August 2024 campaign; Table S9: BaPEq and TEQ for the analyzed sediment samples of October 2023 campaign; Table S10: BaPEq and TEQ for the analyzed sediment samples of August 2024 campaign.

## Abbreviations

The following abbreviations are used in this manuscript:

PAHs	Persistent organic pollutants
PCBs	Polychlorinated biphenyls
OCPs	Organochlorine pesticides
ERL	Effects-range low
ERM	Effects-range median
TEFs	Toxic equivalency factors
ƩPCBs	Total Polychlorinated biphenyls
ƩDDTs	Total Dichlorodiphenyl trichloroethane class
POPs	Persistent organic pollutants
USAE	Ultrasound-assisted extraction
GC	Gas chromatography
EPA	Environmental Protection Agency
HCHs	Hexa-chloro-cyclohexanes
DDTs	Dichlorodiphenyl trichloroethanes
DDE	Dichlorodiphenyl dichloroethylene
DDD	Dichlorodiphenyl dichloroethane
TEC	Threshold effect concentration
PEC	Probable effect concentration
GC-MS	Gas chromatography coupled with mass spectrometry
GC-ECD	Gas chromatography coupled with electron capture detector
NAP	Naphthalene
ACY	Acenaphthylene
ACE	Acenaphthene
FL	Fluorene
PHE	Phenanthrene
ANT	Anthracene
FLT	Fluoranthene
PYR	Pyrene
BaA	Benz[*a*]anthracene
CRY	Chrysene
BbF	Benzo[*b*]fluoranthene
BkF	Benzo[*k*]fluoranthene
BaP	Benzo[a]pyrene
DBahA	Dibenz[*a,h*]anthracene
BghiP	Benzo[*g,h,i*]perylene
IPYR	Indeno[*1,2,3-cd*]pyrene
SIM	Selected Ion Monitoring
SQGs	Sediment quality guidelines
dw	Dry weight
nd	Not detected
LMW	Low molecular weight
HMW	High molecular weight
m-ERM-Q	Mean effect range median quotient
<LOD	Lower than the detection limit
BaP_Eq_	Benzo[a]pyrene equivalent concentration
