# Supplementary material for: POPs Contamination of Aquatic Sediments of the Cerna River Watershed, Romania—Prevalence, Pollution Sources, and Ecological Risk Assessment
Source: Toxics. 2026 Oct 14;14(10):908. doi: 10.3390/toxics14100908 (PMC13709422; doi:10.3390/toxics14100908)
Supplement: Supplementary file 1 [file toxics-14-00908-s001.zip › toxics-4573999-supplementary.pdf]

Supplementary materials

POPs contamination of aquatic sediments of the Cerna River watershed, Romania—prevalence, pollution sources, and ecological risk assessment

Irina Monica Simó [1], Vlad Alexandru Pănescu [2*], Mihaela Cătălina Popovici [2], Ye Yang [1,3], Jérome Vial [4], Maria Concetta Bruzzoniti [5], Mihail Simion Beldean-Galea [1*]

[1] Faculty of Environmental Science and Engineering, Babeș-Bolyai University, 30 Fântânele street, 400294. Cluj-Napoca, Romania
[2] Raluca Ripan Institute for Research in Chemistry, Babeș-Bolyai University, 30 Fântânele street, 400294. Cluj-Napoca, Romania
[3] Key Laboratory of Recycling and Eco-treatment of Waste Biomass of Zhejiang Province, School of Environmental and Natural Resources, Zhejiang University of Science and Technology, Hangzhou, 310023, Zhejiang, China
[4] École Supérieure de Physique et de Chimie Industrielle de la Ville de Paris, Paris Sciences et Lettres University, 10 Rue Vauquelin, 75231 Paris Cedex 05, France
[5] University of Turin, Department of Chemistry, via P. Giuria 5, 10125, Turin, Italy
* Correspondence: simion.beldean@ubbcluj.ro; vlad.panescu@ubbcluj.ro

Table S1. Geographical position of the sampling points

Sample	Location	Coordinates	
		Latitudinal (N)	Longitudinal (E)
**P1**	Cerna River at the confluence with Mureș River	45°52'16.16''	22°57'23.08''
**P2**	Cerna River in Sântuhalm village	45°51'22.10''	22°57'26.11''
**P3**	Cristur River in Cristur village	45°49'30.55''	22°56'29.17''
**P4**	Peștiș River in Peștișul Mare village	45°48'00.45''	22°55'49.95''
**P5**	Petac River in Peștișul Mic village	45°47'46.21''	22°55'10.69''
**P6**	Zlaști River in Zlaști village	45°44'56.77''	22°53'14.30''
**P7**	Teliuc River in Teliucul Inferior village	45°44'04.27''	22°49'26.19''
**P8**	Govâjdie River in Govâjdia village	45°42'56.11''	22°53'16.67''
**P9**	Toplița River in Toplița village	45°41'10.93''	22°47'00.28''
**P10**	Vălarița River in Vălari village	45°41'08.38''	22°46'23.57''

Table S2. TEFs for individual PAHs [45]

Compound	TEF
Naphthalene	0.001
Acenaphthylene	0.001
Acenaphthene	0.001
Fluorene	0.001
Phenanthrene	0.001
Anthracene	0.01
Fluoranthene	0.001
Pyrene	0.001
Benz[*a*]anthracene	0.1
Crysene	0.01
Benzo[*b*]fluoranthene	0.1
Benzo[*k*]fluoranthene	0.1
Benzo[*a*]pyrene	1
Indeno[*123-cd*]pyrene	0.01
Dibenzo[*a,h*]anthracene	1
Benzo[*g,h,i*]perylene	0.1

Table S3. Total PAHs concentration (ng/g dw) and diagnostic ratios for the sources of PAHs for October 2023 campaign.

**Compound**	**1**	**2**	**3**	**4**	**5**	**6**	**7**	**8**	**9**	**10**
Naphthalene	17.47	4.23	8.93	0.98	1.92	1.36	1.69	10.67	76.19	2.80
Acenaphthylene	3.03	0.63	15.57	0.89	0.13	0.74	0.85	0.97	80.10	8.75
Acenaphthene	4.55	1.57	4.52	0.58	0.45	0.72	1.03	2.17	70.76	2.88
Fluorene	3.97	0.29	5.45	0.34	0.20	0.91	0.79	0.87	103.24	10.80
Phenanthrene	31.19	7.59	59.94	7.59	1.79	5.39	10.94	7.72	645.06	89.52
Anthracene	7.69	1.25	40.88	1.53	0.24	1.70	2.72	1.45	243.46	35.28
Fluoranthene	67.77	18.41	198.16	20.43	2.08	10.45	25.18	13.57	949.19	132.10
Pyrene	60.17	16.58	185.78	17.52	1.81	9.29	21.87	12.89	739.92	94.20
Benzo[*a*]anthracene	29.99	8.33	136.22	6.16	0.76	3.41	7.82	4.71	307.86	26.01
Chrysene	35.67	14.68	78.82	8.37	1.11	3.99	9.78	7.73	266.48	22.96
Benzo[*b*]fluoranthene	34.73	16.77	141.28	7.69	1.13	4.43	6.91	8.42	291.51	26.33
Benzo[*k*]fluoranthene	12.66	5.90	34.62	2.78	0.32	1.39	2.68	3.55	112.64	7.43
Benzo[*a*]pyrene	17.41	9.11	68.97	3.80	0.56	2.71	5.04	4.30	215.95	18.42
Benzo[*g,h,i*]perylene	17.64	7.90	59.74	4.21	0.82	2.21	4.21	3.51	144.97	15.77
Indeno[*1,2,3-cd*]pyrene	21.77	10.88	92.04	5.60	1.00	2.85	5.26	5.03	176.33	18.37
Dibenzo[*a,h*]anthracene	6.80	2.46	14.34	1.02	0.31	0.83	1.05	1.57	27.08	2.74
**Total**	**372.53**	**126.57**	**1145.24**	**89.51**	**14.65**	**52.40**	**107.82**	**89.14**	**4450.75**	**514.36**
**∑LMW/∑HMW**	**0.22**	**0.14**	**0.13**	**0.15**	**0.48**	**0.26**	**0.20**	**0.37**	**0.38**	**0.41**
**ANT/(ANT+PHE)**	**0.20**	**0.14**	**0.41**	**0.17**	**0.12**	**0.24**	**0.20**	**0.16**	**0.27**	**0.28**
**FLT/(FLT+PYR)**	**0.53**	**0.53**	**0.52**	**0.54**	**0.53**	**0.53**	**0.54**	**0.51**	**0.56**	**0.58**

Table S4. Total PAHs concentration (ng/g dw) and diagnostic ratios for the sources of PAHs for August 2024 campaign.

**Compound**	**1**	**2**	**3**	**4**	**5**	**6**	**7**	**8**	**9**	**10**
Naphthalene	3.76	1.91	1.93	0.21	0.42	0.30	0.37	2.29	16.48	0.61
Acenaphthylene	0.75	0.29	3.88	0.22	0.03	0.19	0.21	0.24	20.00	2.21
Acenaphthene	1.23	0.76	1.23	0.16	0.12	0.20	0.28	0.59	19.22	0.79
Fluorene	0.88	0.13	1.20	0.08	0.04	0.20	0.17	0.19	22.86	2.41
Phenanthrene	7.50	3.92	14.46	1.86	0.43	1.31	2.64	1.85	155.90	21.85
Anthracene	1.84	0.60	9.84	0.37	0.06	0.41	0.65	0.35	58.67	8.58
Fluoranthene	14.58	7.63	42.79	4.47	0.45	2.27	5.44	2.91	205.29	28.85
Pyrene	13.91	7.06	43.11	4.12	0.42	2.17	5.08	2.98	171.94	22.10
Benzo[*a*]anthracene	8.00	3.20	36.47	1.67	0.20	0.92	2.10	1.25	82.55	7.04
Chrysene	10.99	6.53	24.38	2.62	0.35	1.24	3.03	2.38	82.56	7.18
Benzo[*b*]fluoranthene	10.26	6.49	41.90	2.31	0.34	1.32	2.05	2.48	86.58	7.89
Benzo[*k*]fluoranthene	3.97	2.28	10.89	0.89	0.10	0.44	0.85	1.11	35.49	2.36
Benzo[*a*]pyrene	5.03	3.33	20.00	1.12	0.16	0.79	1.46	1.24	62.71	5.40
Benzo[*g,h,i*]perylene	5.59	3.46	18.99	1.36	0.26	0.71	1.34	1.11	46.16	5.07
Indeno[*1,2,3-cd*]pyrene	7.80	5.24	33.11	2.04	0.36	1.03	1.89	1.80	63.53	6.68
Dibenzo[*a,h*]anthracene	2.32	1.11	4.91	0.36	0.11	0.29	0.36	0.53	9.29	0.95
**Total**	**98.39**	**53.95**	**309.10**	**23.85**	**3.86**	**13.77**	**27.92**	**23.32**	**1139.23**	**129.99**
**∑LMW/∑HMW**	**0.19**	**0.16**	**0.12**	**0.14**	**0.40**	**0.23**	**0.18**	**0.31**	**0.35**	**0.39**
**ANT/(ANT+PHE)**	**0.20**	**0.13**	**0.40**	**0.17**	**0.12**	**0.24**	**0.20**	**0.16**	**0.27**	**0.28**
**FLT/(FLT+PYR)**	**0.51**	**0.52**	**0.50**	**0.52**	**0.52**	**0.51**	**0.52**	**0.49**	**0.54**	**0.57**

Table S5. Total OCPs concentrations (ng/g dw) and molecular ratios for the sources of OCPs for October 2023 campaign.

**Compound**	**1**	**2**	**3**	**4**	**5**	**6**	**7**	**8**	**9**	**10**
Tecnazene	-	-	-	-	-	-	0.81	-	-	-
α-HCH	1.33	1.03	1.42	-	2.51	-	0.63	-	-	3.18
Hexachlorobenzene	0.60	0.27	2.60	-	-	4.15	0	-	-	3.20
β-HCH	4.52	4.25	6.46	-	-	2.94	1.78	2.21	2.48	-
γ-HCH	-	-	10.04	-	-	-	-	-	-	-
Quintozene	-	-	-	7.18	-	6.23	12.71	9.18	12.20	-
Heptachlor	-	5.41	-	-	-	-	-	-	-	-
Aldrin	-	0.72	10.50	0.97	6.36	10.07	-	1.01	0.69	18.92
Heptachlor-Exo	-	-	-	-	-	-	-	-	-	13.84
Heptachlor-Endo	-	-	-	-	-	-	-	-	-	-
Trans-Chlordane	-	-	-	-	-	-	-	-	-	-
2,4´-DDE	-	-	-	-	-	-	-	-	-	-
A-endosulfan	-	-	-	-	-	-	-	-	-	-
Cis-Chlordane	1.01	-	7.91	-	2.53	2.83	2.51	-	-	5.42
Dieldrin	23.45	25.83	8.35	1.80	3.51	7.70	10.20	4.27	4.69	8.02
4,4´-DDE	2.05	4.09	-	-	-	-	5.21	10.10	-	-
2,4´-DDD	1.55	1.51	25.54	-	-	13.63	3.48	-	5.02	27.44
Endrin	-	-	-	-	-	-	-	-	-	-
B-endosulfan	-	-	-	-	-	-	-	-	-	-
4,4´-DDD	2.33	2.31	-	2.30	-	-	-	-	12.54	-
2,4´-DDT	-	-	-	-	-	-	-	-	-	-
4,4´-DDT	6.59	2.49	-	1.37	-	-	-	-	3.21	-
Methoxichlor	-	-	-	-	-	-	-	-	-	-
**Total**	**43.41**	**47.90**	**72.82**	**13.62**	**14.90**	**47.55**	**37.32**	**26.77**	**40.83**	**80.01**
**∑DDTs/∑HCHs**	**2.14**	**1.97**	**1.42**	**-**	**-**	**4.64**	**3.61**	**4.57**	**8.38**	**8.62**
**(DDE+DDD)/∑DDTs**	**0.47**	**0.76**	**1**	**0.63**	**-**	**1**	**1**	**1**	**0.85**	**1**
***α*-HCH/β-HCH**	**0.29**	**0.24**	**0.22**	**-**	**-**	**-**	**-**	**-**	**-**	**-**

"-" not detected/not determined

Table S6. Total OCPs concentrations (ng/g dw) and molecular ratios for the sources of OCPs for August 2024 campaign.

**Compound**	**1**	**2**	**3**	**4**	**5**	**6**	**7**	**8**	**9**	**10**
Tecnazene	-	-	-	-	-	-	0.31	-	-	-
α-HCH	0.66	0.42	1.15	-	1.95	-	1.56	-	-	1.12
Hexachlorobenzene	-	-	16.13	-	1.28	-	-	-	-	-
β-HCH	1.48	1.90	2.48	7.89	-	2.01	9.82	2.45	4.69	-
γ-HCH	-	0	3.2	-	-	-	-	-	-	-
Quintozene	-	21.09	-	15.27	-	-	-	28.47	15.55	-
Heptachlor	-	0	-	0	-	-	-	-	-	-
Aldrin	-	1.12	-	7.31	-	1.99	0.81	5.07	3.17	0.91
Heptachlor-Exo	-	12.01	-	-	-	-	0.53	-	-	1.07
Heptachlor-Endo	-	-	-	-	-	-	-	-	-	-
Trans-Chlordane	-	-	-	-	-	-	-	-	-	-
2,4´-DDE	-	-	-	-	-	-	-	-	-	-
A-endosulfan	-	-	-	-	-	-	-	-	-	-
Cis-Chlordane	-	10.84	-	-	-	-	1.47	-	-	1.15
Dieldrin	2.73	2.24	62.55	4.17	16.67	3.93	1.65	-	-	-
4,4´-DDE	0.25	1.56	0	-	-	-	4.23	6.98	-	-
2,4´-DDD	7.30	5.55	24.11	-	-	2.55	-	-	-	-
Endrin	-	-	-	-	-	-	-	-	-	-
B-endosulfan	-	-	-	-	-	-	-	-	-	-
4,4´-DDD	2.14	-	-	-	3.25	-	-	-	8.95	-
2,4´-DDT	-	-	19.94	-	-	-	-	-	-	-
4,4´-DDT	5.36	1.95	-	-	-	-	-	-	0.62	-
Methoxichlor	-	-	-	-	-	-	-	-	-	-
**Total**	**19.91**	**58.69**	**129.56**	**34.63**	**23.15**	**10.48**	**20.37**	**42.96**	**32.97**	**4.25**
**∑DDTs/∑HCHs**	**7.04**	**3.90**	**6.45**	**-**	**1.67**	**1.27**	**0.37**	**-**	**2.04**	**-**
**(DDE+DDD)/∑DDTs**	**0.64**	**0.78**	**0.55**	**-**	**1**	**1**	**1**	**1**	**0.94**	**-**
**α-HCH/β-HCH**	**0.45**	**0.22**	**0.47**	**-**	**-**	**-**	**0.16**	**-**	**-**	**-**

"-" not detected/not determined

Table S7. PCBs concentrations for October 2023 campaign.

Compound	1	2	3	4	5	6	7	8	9	10
PCB -18	-	-	-	12.46	-	5.24	4.85	-	-	8.59
PCB - 31+28	0.81	-	-	14.91	-	-	4.19	-	-	6.58
PCB -52	2.93	5.23	9.53	-	4.71	5.64	4.12	5.00	2.06	-
PCB -44	-	-	-	1.89	-	-	-	-	-	3.17
PCB -101	-	-	3.87	-	-	3.43	-	-	-	7.33
PCB -114	-	-	3.59	3.01	-	-	-	-	-	2.82
PCB -149	-	-	2.68	-	-	-	3.32	-	-	1.75
PBC -153	-	-	7.79	-	-	2.01	-	-	-	4.45
PCB -138+180	-	-	4.01	-	-	-	-	-	-	6.12
PCB -194	-	-	-	-	-	-	-	-	-	-
**Total (ng/g)**	**3.74**	**5.23**	**31.47**	**32.26**	**4.71**	**16.32**	**16.48**	**5.00**	**2.06**	**40.81**

"-" not detected

Table S8. PCBs concentrations for August 2024 campaign.

Compound	1	2	3	4	5	6	7	8	9	10
PCB -18	-	-	-	5.96	-	2.35	4.11	-	-	7.16
PCB - 31+28	0.7	-	-	9.86	-	-	3.18	-	-	5.98
PCB -52	2.33	-	7.26	-	3.68	3.53	2.24	4.96	0.89	7.40
PCB -44	-	-	-	0.25	-	-	-	-	-	0.56
PCB -101	-	-	-	-	-	-	-	-	-	-
PCB -114	-	-	-	-	-	-	-	-	-	2.23
PCB -149	0.65	-	2.02	-	-	-	2.87	-	-	1.74
PBC -153	1.56	-	3.56	-	-	0.26	-	-	-	1.09
PCB -138+180	-	-	1.46	-	-	-	-	-	-	2.92
PCB -194	-	-	-	-	-	-	-	-	-	-
**Total (ng/g)**	**5.24**	**-**	**14.31**	**16.07**	**3.68**	**6.14**	**12.40**	**4.96**	**0.89**	**29.07**

"-" not detected

Table S9. BaP$_{Eq}$ and TEQ for the analyzed sediment samples of October 2023 campaign.

Compound	Sample										
	**TEF***	**1**	**2**	**3**	**4**	**5**	**6**	**7**	**8**	**9**	**10**
	**BaP$_{Eq}$**										
NAP	*0.001*	0.017	0.004	0.009	0.001	0.002	0.001	0.002	0.011	0.076	0.003
ACY	*0.001*	0.003	0.001	0.016	0.001	-	0.001	0.001	0.001	0.080	0.009
ACE	*0.001*	0.005	0.002	0.005	0.001	-	0.001	0.001	0.002	0.071	0.003
FL	*0.001*	0.004	-	0.005	-	-	0.001	0.001	0.001	0.103	0.011
PHE	*0.001*	0.031	0.008	0.060	0.008	0.002	0.005	0.011	0.008	0.645	0.090
ANT	*0.01*	0.077	0.013	0.409	0.015	0.002	0.017	0.027	0.014	2.435	0.353
FLU	*0.001*	0.068	0.018	0.198	0.020	0.002	0.010	0.025	0.014	0.949	0.132
PYR	*0.001*	0.060	0.017	0.186	0.018	0.002	0.009	0.022	0.013	0.740	0.094
BaA	*0.1*	2.999	0.833	13.622	0.616	0.076	0.341	0.782	0.471	30.786	2.601
CRY	*0.01*	0.357	0.147	0.788	0.084	0.011	0.040	0.098	0.077	2.665	0.230
BbF	*0.1*	3.473	1.677	14.128	0.769	0.113	0.443	0.691	0.842	29.151	2.633
BkF	*0.1*	1.266	0.590	3.462	0.278	0.032	0.139	0.268	0.355	11.264	0.743
BaP	*1*	17.409	9.106	68.973	3.803	0.559	2.710	5.037	4.301	215.949	18.421
IPYR	*0.01*	0.218	0.109	0.920	0.056	0.010	0.029	0.053	0.050	1.763	0.184
DBahA	*1*	6.797	2.460	14.336	1.024	0.306	0.835	1.046	1.566	27.081	2.743
BghiP	*0.1*	1.764	0.790	5.974	0.421	0.082	0.221	0.421	0.351	14.497	1.577
**TEQ**	**NA**	**34.548**	**15.773**	**123.091**	**7.114**	**1.200**	**4.803**	**8.486**	**8.078**	**338.255**	**29.825**
**ΣCPAHs**	**NA**	**31.284**	**14.878**	**108.582**	**6.434**	**1.114**	**4.416**	**7.614**	**7.544**	**302.370**	**26.530**
% DBahA		21.73	16.54	13.20	15.91	27.44	18.90	13.74	20.76	8.96	10.34
% BaP		55.65	61.20	63.52	59.11	50.21	61.36	66.15	57.02	71.42	69.44

Carcinogenic PAHs; Non carcinogenic PAHs; *TEF values proposed by Nisbet and Lagoy (1992); "-" not calculated; NA—not assigned

Table S10. BaP$_{Eq}$ and TEQ for the analyzed sediment samples of August 2024 campaign.

Compound	TEF*	1	2	3	4	5	6	7	8	9	10
		**Sample**									
		**BaP$_{Eq}$**									
NAP	*0.001*	0.004	0.002	0.002	-	-	-	-	0.002	0.016	0.001
ACY	*0.001*	0.001	-	0.004	-	-	-	-	-	0.020	0.002
ACE	*0.001*	0.001	0.001	0.001	-	-	-	-	0.001	0.019	0.001
FL	*0.001*	0.001	-	0.001	-	-	-	-	-	0.023	0.002
PHE	*0.001*	0.007	0.004	0.014	0.002	-	0.001	0.003	0.002	0.156	0.022
ANT	*0.01*	0.018	0.006	0.098	0.004	0.001	0.004	0.007	0.003	0.587	0.086
FLU	*0.001*	0.015	0.008	0.043	0.004	-	0.002	0.005	0.003	0.205	0.029
PYR	*0.001*	0.014	0.007	0.043	0.004	-	0.002	0.005	0.003	0.172	0.022
BaA	*0.1*	0.800	0.320	3.647	0.167	0.020	0.092	0.210	0.125	8.255	0.704
CRY	*0.01*	0.110	0.065	0.244	0.026	0.003	0.012	0.030	0.024	0.826	0.072
BbF	*0.1*	1.026	0.649	4.190	0.231	0.034	0.132	0.205	0.248	8.658	0.789
BkF	*0.1*	0.397	0.228	1.089	0.089	0.010	0.044	0.085	0.111	3.549	0.236
BaP	*1*	5.028	3.333	19.999	1.117	0.163	0.790	1.462	1.241	62.709	5.401
IPYR	*0.01*	0.078	0.052	0.331	0.020	0.004	0.010	0.019	0.018	0.635	0.067
DBahA	*1*	2.319	1.111	4.911	0.355	0.105	0.288	0.359	0.534	9.291	0.950
BghiP	*0.1*	0.559	0.346	1.899	0.136	0.026	0.071	0.134	0.111	4.616	0.507
**TEQ**	**NA**	**10.378**	**6.134**	**36.518**	**2.156**	**0.368**	**1.449**	**2.523**	**2.427**	**99.737**	**8.892**
**ΣCPAHs**	**NA**	**9.517**	**5.785**	**32.664**	**1.974**	**0.345**	**1.347**	**2.293**	**2.287**	**90.284**	**8.023**
% DBahA		24.37	19.21	15.04	17.99	30.45	21.35	15.64	23.34	10.29	11.84
% BaP		52.83	57.62	61.23	56.59	47.16	58.65	63.74	54.25	69.46	67.32

Carcinogenic PAHs; Non carcinogenic PAHs; *TEF values proposed by Nisbet and Lagoy (1992); "-" not calculated; NA—not assigned
